# Efficacy and safety of mycophenolate mofetil treatment in IgA nephropathy: a systematic review

**DOI:** 10.1186/1471-2369-15-193

**Published:** 2014-12-05

**Authors:** Youyuan Chen, YuMin Li, ShengLin Yang, Yan Li, Min Liang

**Affiliations:** Division of Nephrology, Nanfang Hospital, Southern Medical University, Guangzhou, Guangdong China

**Keywords:** IgA nephropathy, Mycophenolate mofetil, Immunosuppressant, Systematic review

## Abstract

**Background:**

IgA nephropathy is the most common primary glomerular disease worldwide and also the most frequent cause of kidney failure. Mycophenolate mofetil (MMF) is a selective immunosuppressant widely used in many autoimmune diseases. However, the benefits and risks of MMF for the treatment of IgA nephropathy remain uncertain.

**Methods:**

A systematic review and meta-analysis of randomized controlled trials (RCTs) was performed to assess the efficacy and safety of MMF in IgA nephropathy patients, using the statistical software Review Manager 5.1.

**Results:**

Eight RCTs involving 357 patients were identified and included in this review. Overall, no statistical difference was found in the therapeutic effect of MMF treatment compared with other therapies. MMF had no significant effects on reducing proteinuria or protecting renal function. However, subgroup analysis indicated that relatively short-term therapy (<18 months) might be beneficial in IgA nephropathy patients while longer term MMF use conferred no advantage. There was also no statistical difference between MMF and control groups in the incidence of side effects. When compared with other immunosuppressants, MMF was considered superior to cyclophosphamide in terms of better therapeutic effects and fewer adverse reactions, but no difference was found between MMF and leflunomide.

**Conclusions:**

Our current evidence indicates that a relatively short course of MMF may be beneficial in treating IgA nephropathy. However, high-quality RCTs with large sample size as well as a well-designed study to evaluate the long-term effects of MMF are needed to further evaluate the efficacy and safety of MMF in this disease.

**Electronic supplementary material:**

The online version of this article (doi:10.1186/1471-2369-15-193) contains supplementary material, which is available to authorized users.

## Background

Immunoglobulin A nephropathy is the most common type of glomerulonephritis in the world [1, 2] and causes end-stage renal disease (ESRD) in a significant percentage of patients [3–5]. About 1–2% of patients who are newly diagnosed with IgA nephropathy will develop ESRD each year [6]. Some clinical markers such as impaired kidney function, sustained hypertension, and heavy proteinuria (over 1 g per day) are associated with poor prognosis [7–9]. Most of the current treatment strategies including blood pressure (BP) control, angiotensin converting enzyme inhibitors (ACEI) or angiotensin receptor blockers (ARB), lead to a reduction of proteinuria and are also commonly used in patients suffering from other chronic kidney diseases. However, there is still no specific treatment to date available for IgA nephropathy because its pathogenic mechanisms remain incompletely understood.

Mycophenolate mofetil (MMF), which is used as an immunosuppressant in patients undergoing renal transplants, might be an option for immunosuppressive treatment of patients with autoimmune diseases [10, 11]. However, the efficacy of MMF therapy in IgA nephropathy is controversial. Several reviews evaluated the use of MMF in IgA nephropathy patients but they were published too early to include novel trials or lacked sufficient safety evaluations [12–14].

In this systematic review, we sought all available randomized controlled trials (RCTs) to comprehensively evaluate the efficacy and safety of MMF therapy in IgA nephropathy.

## Methods

### Data sources and search strategy

We searched electronic databases of MEDLINE, EMBASE, the Cochrane Library, Chinese Biomedical Literature Database (CBM), and China National Knowledge Infrastructure (CNKI) up to August 2014 with relevant key words and medical subject headings covering IgA nephropathy, IgA, GN, IgAGN, Berger’s disease, mycophenolate mofetil, mycophenolic acid, MMF, CellCept, controlled clinical trial, RCT, randomized controlled trial, and drug therapy without any language restriction (Additional file 1). Studies were excluded if they were not RCTs or had a follow-up period of less than 6 months. Manual scanning of reference lists from identified trials and review articles was done to identify any further studies that may have been relevant. All our work in this systematic review referred to the PRISMA (Preferred Reporting Items for Systematic Reviews and Meta-Analyses) guidelines (Additional file 2) [15]. All analyses were based on previous published studies, thus no ethical approval and patient consent are required.

### Study selection criteria

We included studies with biopsy-proven IgA nephropathy patients, aged ≥18 years old, with daily proteinuria ≥ 1 g but no malignancy and those studies comparing the efficacy of MMF with control or other immunosuppressive agents.

Studies that were not RCTs, or those with follow-up periods of less than 6 months were excluded.

### Data extraction and quality assessment

Information from each trial was separately extracted by two authors (Chen YY and Li YM) using standard data-extraction forms. The extracted data included the baseline information of participants, proteinuria level, doses and duration of MMF use, follow-up duration, clinical outcomes, and adverse events.

Regarding any study with several publications, all the reports were grouped together. For some, information that was not reported in the publication or was presented in diagrams only, the investigators contacted the authors to seek the missing data.

Two additional authors (Yang SL and Li Y) who were not blind to authorship or journal of publication were responsible for the study quality, which included allocation concealment, blinding and completeness of follow-up [16].

### Outcome measures

The primary outcome was therapeutic effect. This included complete remission, defined as a value for urinary protein excretion that was below 0.3 g/24 h and a normal serum creatinine (Scr) level; significant remission, defined as a decline in urinary protein excretion by 50% or more over baseline value and a decline of Scr by 20% or more; partial remission, defined as decline in urinary protein excretion between 30 and 50%, as well as a relatively stable Scr level (variation less than 20%); treatment failure, defined as those failing to meet the standards outlined above.

The rate of therapeutic effect was calculated using the equation: effective rate = complete remission rate + significant remission rate + partial remission rate.

Secondary outcomes were ESRD, 50% increase in Scr, reduction of proteinuria and adverse effects.

### Statistical analysis

Analyses were performed using Review Manager 5.1. The results of dichotomous outcomes (therapeutic effects, ESRD events, doubling Scr increases and adverse reaction) were expressed as risk ratios (RR) with 95% confidence intervals (CI). The mean difference (MD) was obtained when continuous scales of measurement were used to assess the treatment effects (e.g. proteinuria) and the standardized mean difference (SMD) was obtained when different scales were used. Inter-study heterogeneity was assessed by using the chi-square test and subgroup analysis was performed when high heterogeneity between studies existed. A random-effects model was used in this study because such models are generally considered to be more conservative [17].

## Results

### Search results

**Figure 1:**
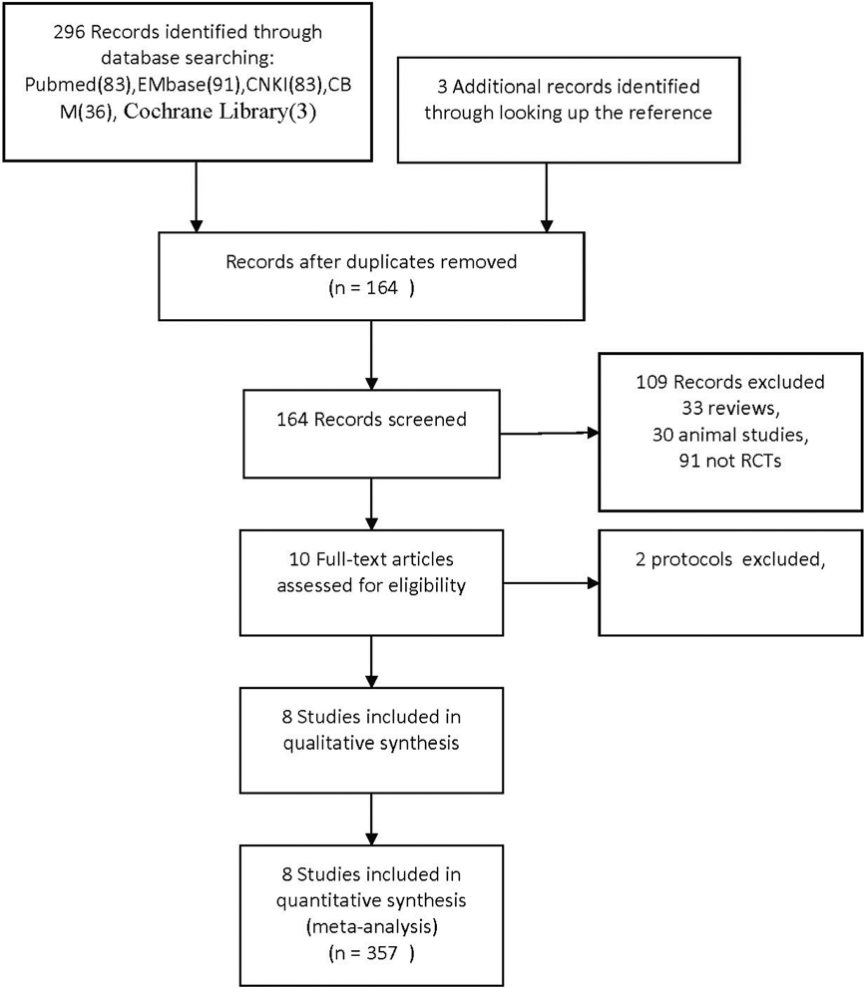
**Flow diagram of study selection.**

A comprehensive literature search identified 164 articles after removing duplicates, 91 of which did not involve RCTs and were thus excluded. Animal studies were also excluded. The full texts of ten articles were analyzed, and an additional two were excluded because they detailed protocols only [18, 19]. Finally, eight articles [20–27] were identified and retained for this review. These eight studies involved 357 patients, 190 of which were in the MMF treatment group and 167 in the control groups (48 received placebo, 31 received steroid treatment, 68 received cyclophosphamide [CTX], and 20 received leflunomide [LEF]; Figure 1).

### Characteristics of the included studies

The characteristics of the included studies are summarized in Table 1. All the included studies were prospective RCTs comparing the efficiency of MMF with controls. Other treatment regimens included antihypertensive agents (Calcium channel blocker (CCB)/ACEI/ARB) for BP control and antiplatelet drugs when necessary. The placebo group was not treated with any steroids or immunosuppressants. Two studies [19, 23] included patients with histologically unfavorable criteria (grade V). One study [24] included patients with crescentic IgA nephropathy and a further study included patients with nephritic syndrome [26]. All eight studies included patients with proteinuria over 1 g/d. Scr levels of patients in all eight studies ranged from 1.04 mg/ml to 2.6 mg/ml but the mean estimated glomerular filtration rate (eGFR) of patients was not available.

**Table 1:** **Characteristics of RCTs involved in the study**

Study	Patients	Scr ^a^(mg/dl)	Proteinuria ^a^(g/d)	Systolic BP (mmHg)	Sample size ^b^	MMF		Control	Follow–up (m)	Drop-in/MMF discontinuation	Other treatment regimen
						Dose (g/d)	Duration (m)				
**Chen et al. 2002** [20]	Grades:IV-V with interstitial inflammation area > 25%; Proteinuria > 2 g/d	NA	MMF:3.2 ± 1.7; Control:2.9 ± 1.5	NA	62 (31/31)	1.0–1.5	12	Steroid	18	NA/0	CCB, antiplatelet when needed
**Meas et al. 2004** [21]	Grades:II-IV; Proteinuria > 1 g/d; Icr > 20 but < 70 mL/min/1.73 m^2^	MMF:1.46 ± 0.08 Control: 1.39 ± 0.1	MMF:1.9 ± 0.3; Control:1.3 ± 0.4	MMF:122 ± 4 Control:134 ± 8	34 (21/13)	2	36	Placebo	36	NA/1	Salt restriction, ACEI/ARB or CCB
**Frisch et al. 2005** [22]	Proteinuria > 1 g/d; Ccr > 20 but < 80 ml/min; glomerulosclerosis/tubulointerstitial fibrosis and/or crescent ≥25%	MMF:2.6 ± 1.2 Control:2.2 ± 0.72	MMF:2.7 ± 1.6; Control:2.7 ± 1.4	MMF:136 ± 19.2 Control:131 ± 10.6	32 (17/15)	Up to 2 g/d	12	Placebo	24	NA/0	ACEI/ARB
**Tang et al. 2005** [23]	Grades:II-IV; Proteinuria > 1 g/d;	MMF:1.53 ± 0.17; Control:1.65 ± 0.23	MMF:1.8 ± 0.21; Control:1.87 ± 0.28	MMF:120 ± 3.2 Control:122 ± 3.2	40 (20/20)	1.5–2	6	Placebo	18	NA/0	Salt restriction; ACEI/ARB
**Zhao et al. 2005** [24]	Grades ≥ III	All:1.6 ± 0.53	All:2.03 ± 0.67	NA	31 (21/10)	1.0–1.5	24	CTX	24	2/0	Prednisone 0.6 mg/kg.d in both groups; CCB or ACEI/ARB
**Bao et al. 2007** [25]	Crescentic nephropathy; crescent ≥15%	MMF:1.21 ± 0.96; Control:1.15 ± 0.57	MMF:2.94 ± 2.11; Control:2.87 ± 1.69	NA	34 (18/16)	1.0–1.5	6	CTX	12	NA/0	Methylprednisolone 0.5 g iv for first 3ds, 0.8 mg/kg.d p.o. CCB or ACEI/ARB
**Liu et al. 2010** [26]	Nephritic syndrome	MMF:1.09 ± 0.27; Control:1.04 ± 0.29	MMF:2.6 ± 1.2; Control:2.2 ± 0.72	NA	40 (20/20)	1.5	6	LEF	6	NA/0	Prednisone 0.8 mg/kg.d in both MMF and control;
**Liu et al. 2014** [27]	Proteinuria > 1 g/d; Scr < 3 mg/dl; CrCl > 50/1.73 m^2^ Grade ≥ III	MMF:1.5 ± 0.4 Control:1.4 ± 0.4	MMF:2.83 ± 0.65 Control:2.77 ± 0.81	MMF:141 ± 15.4 Control:134 ± 17.7	84 (42/42)	1.5 g	18	CTX	18	NA/0	Prednisone 0.8–1.0 mg/kg.d in both groups; CCB or ACEI/ARB antiplatelet when needed

Drop in, patients who are randomized to the standard/control arm but start taking/using the experimental treatment; Scr, serum creatinine; NA, not available; Icr, insulin clearance; Ccr, creatine clearance; UPC, urine protein-to-creatinine; CTX, cyclophosphamide; LEF, leflunomide; IQR, interquartile range.

^a^Expressed as mean ± SD or median (IQR).

^b^Expressed as total number of patients (number in steroid group/number in control group).

### Study quality

Many studies provided few details on the method of randomization and the concealment of allocation. Only one mentioned that they adequately concealed allocation. All of the trials were small (31–84 participants) and most of them (86%) used an open-label design (Additional files 3 and 4).

### Heterogeneity among studies

**Figure 2:**
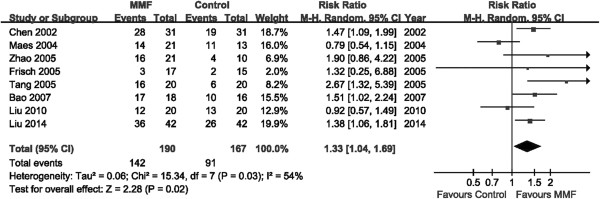
**Heterogeneity among studies.**

The heterogeneity of all the included studies was assessed in Figure 2*.* Treatment regimens in the control groups may be a source of the heterogeneity between the eight included studies (Table 2), so we divided our RCTs into the subgroups ‘MMF vs placebo (or small dose steroid only)’ and ‘MMF vs other immunosuppressants’, to assess the outcome data. Other factors such as duration of MMF use, race and mean proteinuria may also contribute to the high heterogeneity of the four studies comparing MMF to placebo (or small dose of steroid) (Table 3). However, more evidence is needed because of the limited number of RCTs available.

**Table 2:** **Sources of heterogeneity in all studies**

Potential source of heterogeneity	Studies (n)	I ^2^	P value
**Total**	**8**	**54%**	**0.03**
Treatment of Control			
Placebo (or small dose steroid)	4	75%	0.009
Other immunosuppressants	4	13%	0.33

**Table 3:** **Sources of heterogeneity in MMF vs placebo (or small dose of steroid)**

Potential source of heterogeneity	Studies (n)	I ^2^	P value
Placebo (or small dose steroid)	4	75%	0.007
Control			
Small dose of steroid	1	-	-
Placebo	3	82%	0.003
Race			
Asian	2	62%	0.1
Non-Asian	2	0%	0.49
Duration of MMF use			
<18 moths	3	23%	0.27
≥18 months	1	-	-
Mean Proteinuria			
<2 g	2	91%	0.0007
≥2 g	2	0%	0.89

### Outcome

#### Therapeutic effect

**Figure 3:**
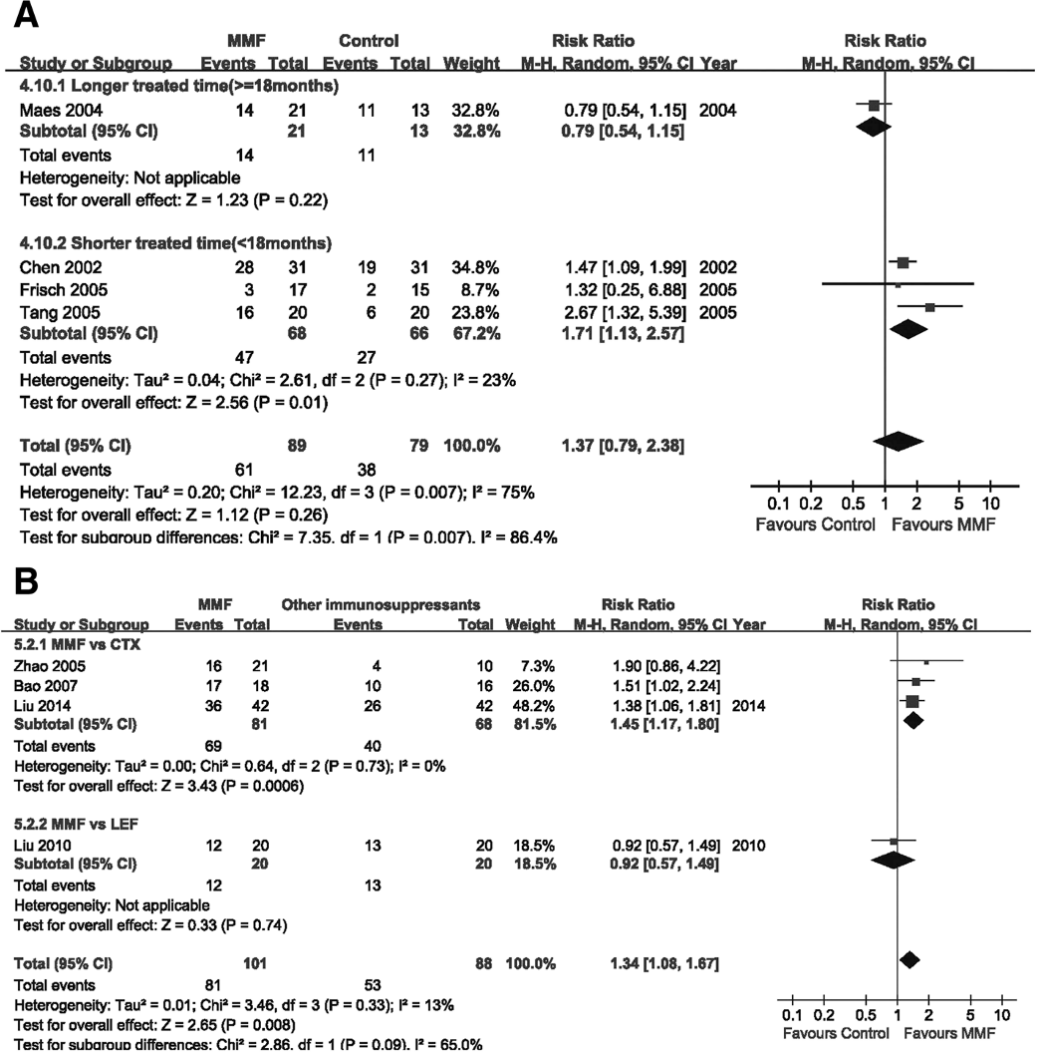
**Forest plot of therapeutic effect of patients treated with MMF or control therapy.** Studies were identified by the year of publication. Risk ratios (RRs) were pooled using the random-effect model. **A**: MMF vs placebo (or steroid). **B**: MMF vs other immunosuppressants.

The therapeutic effect was compared in all eight studies, four of which compared MMF with placebo or steroid [20–23]. The remaining four trials compared MMF with other immunosuppressive agents: three were versus CTX [24, 25, 27] and one was compared with LEF [26]. No difference was observed between the MMF and placebo groups, which comprised four trials, 168 patients, RR: 1.37, 95% CI: 0.79 to 2.38, *P* = 0.26; with significant heterogeneity (I^2^ = 75%; *P* = 0.007; Figure 3A). Better therapeutic effect was shown in the MMF group, encompassing three trials, 149 patients, RR: 1.45, 95% CI: 1.17 to 1.80, *P* = 0.0006; heterogeneity: I^2^ = 0%; *P* = 0.73, than in the CTX group. However, there was no significant difference between the MMF and LEF groups, indicated by one trial covering 40 patients, RR: 0.92, 95% CI: 0.57 to 1.49, *P* = 0.74 (Figure 3B).

Because of the high heterogeneity between the MMF and placebo (or steroid) groups, we performed subgroup analysis according to the duration of MMF use after the comparison of clinical characteristics [20–23]. Our result seemed to suggest that a short MMF treatment time (<18 months) had potential benefits in IgA nephropathy patients (period of <18 months: three trials, 134 patients; RR: 1.71, 95% CI: 1.13 to 2.57, *P* = 0.01; heterogeneity: I^2^ = 23%; *P* = 0.27), while no significant effect was observed in the long-term treatment group (one trial, 34 patients; RR: 0.79, 95% CI: 0.54 to 1.15, *P* = 0.22; Figure 3A).

#### Effect on ESRD

**Figure 4:**
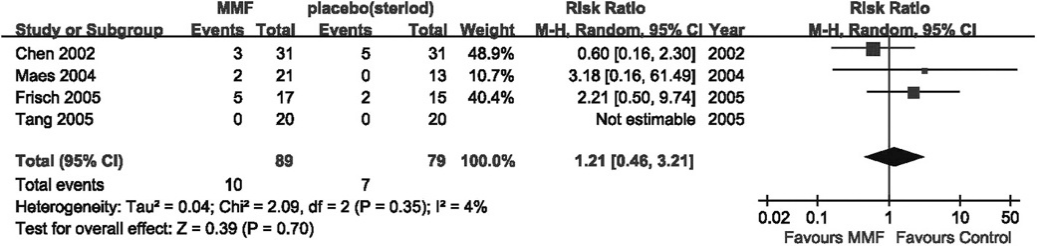
**Forest plot of ESRD in patients treated with MMF or placebo (steroid) therapy.** Studies were identified by the year of publication. Risk ratios (RRs) were pooled using the random-effect model.

All four studies which compared MMF with placebo (or steroid) assessed the need for renal-replacement therapy, covering 168 patients. Ten of the 89 patients in the MMF treatment group and seven of the 79 patients in the control groups required renal-replacement therapy, but this was not statistically significant (RR: 1.21, 95% CI: 0.46 to 3.12, *P* = 0.70; heterogeneity: I^2^ = 4%, *P* = 0.35) versus control (Figure 4).

#### Effect on proteinuria

**Figure 5:**
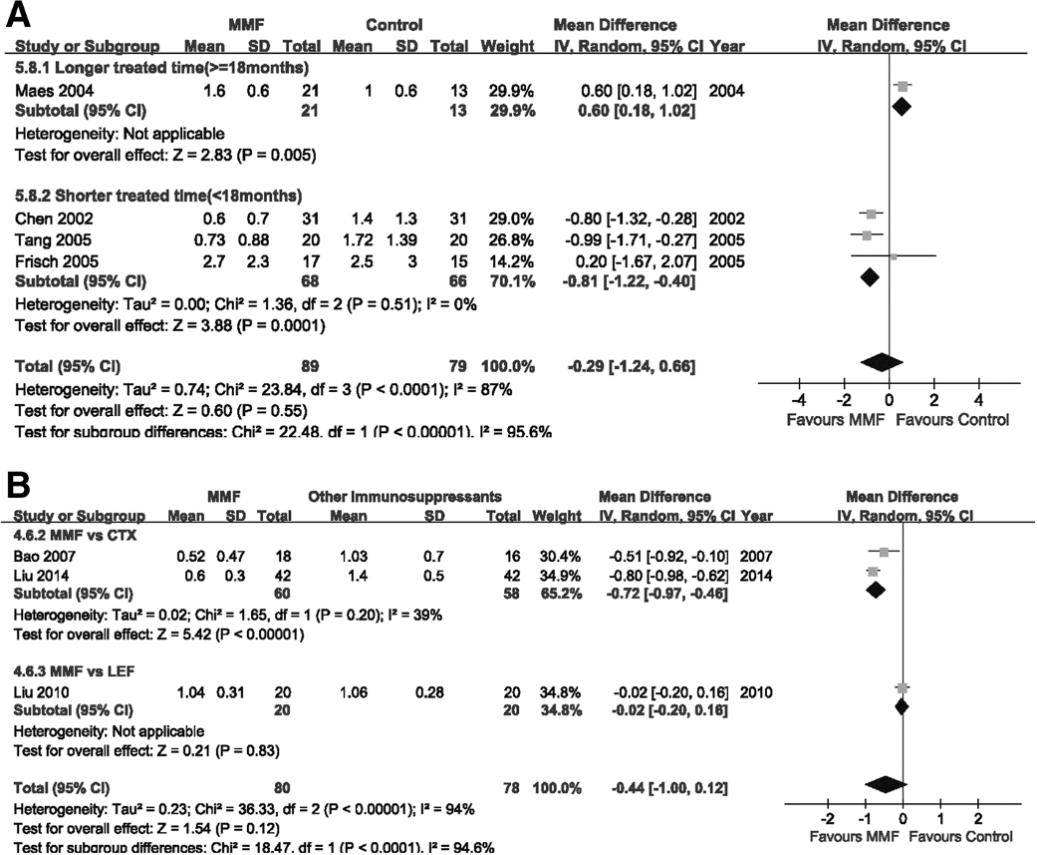
**Forest plot of 24 h proteinuria of patients treated with MMF or control therapy.** Studies were identified by the year of publication. Mean differences (MDs) were pooled using the random-effect model. **A**: MMF vs placebo (or steroid). **B**: MMF vs other immunosuppressants.

Seven studies assessed proteinuria in a total of 326 patients. No difference was observed between the MMF and placebo groups (four trials, 168 patients; MD: -0.29, 95% CI: -1.24 to 0.66, *P* = 0.55; heterogeneity: I^2^ = 87%; *P* = 0.0001; Figure 5A). When comparing the effect on lowering proteinuria, MMF appears to be better than CTX (two trials, MD:-0.72, 95% CI: -0.97 to -0.46, *P* < 0.00001) in lowering the urinary protein level. However, no difference was found in MMF versus LEF groups (one trial, 40 patients, MD: -0.02, 95% CI: -0.20 to 0.16, *P* = 0.83; Figure 5B).

As mentioned above, when performing subgroup analysis according to duration of MMF use, the group receiving shorter MMF treatment (<18 months) benefited from a lower urinary protein level (<18 months: three trials, 134 patients; MD -0.81, 95% CI: -1.22 to -0.40, *P* = 0.0001; heterogeneity: I^2^ = 0%; *P* = 0.51) than those receiving more prolonged treatment (one trial, 34 patients; MD: 0.60, 95% CI: 0.18 to 1.02, *P* = 0.005; Figure 5A).

#### Effect on Scr

**Figure 6:**
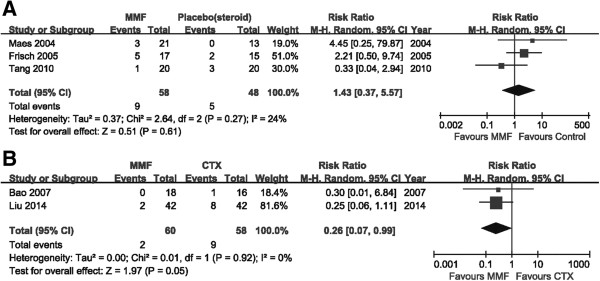
**Forest plot of Scr in patients treated with MMF or control therapy.** Studies were identified by the year of publication. Risk ratios (RRs) were pooled using the random-effect model. **A**: MMF vs placebo (or steroid). **B**: MMF vs CTX.

Five studies assessed the increase of Scr in 224 patients. A 50% increase in Scr was seen in 11 of the 118 patients in the MMF-treated group, and in 14 of the 106 patients in the control groups. There was no statistically significant difference between the MMF-treated group and the controls in the number of patients who achieved a 50% increase in Scr (three trials, 106 patients; RR: 1.43, 95% CI: 0.37 to 5.57, *P* = 0.61; heterogeneity: I^2^ = 24%; *P* = 0.27; Figure 6A). This was also apparent when comparing MMF versus CTX-treated groups (two trials, 118 patients; RR: 0.26, 95% CI: 0.07 to 0.99, *P* = 0.45; heterogeneity: I^2^ = 0%; *P* = 0.92; Figure 6B).

#### Adverse events

Data on adverse outcomes potentially associated with treatment were collected from the trials (Table 4). Several adverse events were reported (gastrointestinal complaints or temporary aminotransferase rise, infection, leucopenia, anemia). No severe infection or liver damage was reported in these trials. Furthermore, there was no difference between MMF and placebo on the incidence of side effects in either the MMF versus CTX or LEF comparison groups. A trend towards an increasing risk of gastrointestinal disorders (RR: 5.02, 95% CI: 0.83 to 30.15, *P* = 0.08) following MMF treatment was observed but did not reach statistical significance.

**Table 4:** **Adverse events reported in the included RCTs**

Adverse events	Studies reporting (n)	MMF (n/n)	Control (n/n)	RR (95% CI)	P value
Total patients with adverse events	7	27/148	20/125	1.14 [0.67, 1.93]	0.63
Specific adverse events					
Infection					
MMF vs Placebo (or Steroid)	3	5/72	0/64	3.84 [0.61, 24.03]	0.15
MMF vs other immunosuppressive agents	2	4/61	5/60	0.77 [0.22, 2.71]	0.68
Gastrointestinal disorders					
MMF vs Placebo (or Steroid)	3	7/72	0/64	5.02 [0.83, 30.15]	0.08
MMF vs other immunosuppressive agents	3	2/81	8/80	0.25 [0.05, 1.13]	0.07
Abnormal liver function					
MMF vs Placebo (or Steroid)	0	0	0	-	-
MMF vs other immunosuppressive agents	4	4/102	6/90	0.66 [0.18, 2.42]	0.74
Abnormal blood counts					
MMF vs Placebo (or Steroid)	2	4/41	0/33	4.25 [0.46, 39.30]	0.20
MMF vs other immunosuppressive agents	3	3/82	5/70	0.53 [0.15, 1.94]	0.34
Hair loss					
MMF vs Placebo (or Steroid)	0	0	0	-	-
MMF vs other immunosuppressive agents	2	0/61	2/60	0.32 [0.03, 3.02]	0.32
Irregular menstruation					
MMF vs Placebo (or Steroid)	0	0	0	-	-
MMF vs other immunosuppressive agents	1	0/19	2/18	0.19 [0.01, 3.71]	0.27

## Discussion

In this systematic review, eight RCTs were included, covering 357 patients in total. No difference was found between MMF and other treatments or controls in the therapeutic effect or the effect on proteinuria, while subgroup analysis indicated that duration of MMF use of less than 18 months may have better therapeutic effects such as a 30–50% decrease in urinary protein as well as a relatively stable or 20% decrease in Scr. The satisfactory tolerance for MMF over a relatively short time course or some unpredictable side effects after longer term use of MMF may explain why a shorter duration of MMF treatment was beneficial. However, only one study, including 34 patients, assessed the effects of longer term therapy with MMF, but its follow-up period was too short. Therefore, based on this observation, the benefits of a short term treatment with MMF are yet to be irrefutably confirmed. No statistically significant difference was found in the MMF group compared with other therapies, either in the need for renal-replacement therapy or in the outcome of 50% increase of Scr. As for the comparison between MMF and other immunosuppressants, MMF may be considered superior to CTX with better clinical therapeutic effect.

When comparing the MMF-treated and control groups, no difference was found either in the effect of ESRD or in the effect of a 50% increase in Scr. However, Sydney Tang et al. [28] extended their original study [23] by following 40 Chinese patients with established IgA nephropathy for 6 years to evaluate the long term effect of MMF treatment. They found two (10%) patients in the MMF group and nine (45%) patients in the control group developed progressive renal failure that required dialysis at the end of this 6-year follow-up. This hints that MMF may have a long term effect of renoprotection. In a self-controlled clinical trial, Dario Roccatello et al. [29] found that parameters including Scr, proteinuria and microscopic hematuria significantly dropped at 6 months and remained lower at the end of a follow-up period of 51 months. In this systematic review, the follow-up period of the included trials was too short (range 6 to 36 months) to detect long term effects of MMF therapy. In some observational studies, MMF combined with low-dose prednisone with duration of around 12–18 months can reduce proteinuria and preserve renal function [30–32]. According to our result, no difference was found in the incidence of side effects. However, delayed side effects such as delayed severe pneumonia could not be ruled out [30].

In our analysis, the serum creatinine level of most patients in all eight studies ranged from 1.04 mg/ml to 2.6 mg/ml, among which one study [20] did not state the exact data for Scr levels, one study [22] included patients with the mean level of Scr over 2 mg/dl but below 2.6 mg/dl and the remaining studies included patients with mean Scr below 1.65 mg/dl. The eGFR (or creatine clearance rate) of all included patients were over 20 mL/min/1.73 m^2^; but below 80 mL/min/1.73 m^2^. There was limited variability in kidney function of patients in all eight studies, so we considered there to be no need for subgroup analysis according to the level of Scr. However, some factors should be taken into account, such as patient ethnicity and the mean proteinuria at baseline. Two studies whose participants were all Asian drew the conclusion that MMF was effective in lowering proteinuria, while two further studies with participants mostly of Caucasian ethnicity held the opposite opinion. These data imply that race may influence the efficacy of MMF treatment in IgA nephropathy. Clinical reports suggest that individuals of Asian/Pacific Island heritage are more likely to be affected by IgA nephropathy than other races and furthermore, IgA nephropathy may have a more severe disease course in certain Asian populations [3, 33]. Unfortunately, with the limited information available from our included studies, we could not undertake subgroup analysis according to patient race.

Earlier systematic reviews [12–14] had drawn the conclusion that no benefits were seen in IgA nephropathy patients treated with MMF. However, by using a more rigorous method, including subgroup analysis to resolve the high heterogeneity among those trials, we may draw a different conclusion. According to the results of subgroup analysis, duration of MMF therapy of less than 18 months may be beneficial. We also attempted to tabulate adverse events following MMF treatment. In addition, we compared the MMF and other immunosuppressive agents (CTX and LEF) with regard to their effectiveness and safety in IgA nephropathy.

However, our systematic review has some limitations. Most of the studies were single center studies with a limited number of patients. The follow-up period of these trials ranged from 6 to 36 months, which was not long enough to detect any long-term effects of MMF treatment in IgA nephropathy. For this reason, large, high-quality multicenter clinical trials with longer follow-up period are urgently needed.

Despite the limitations, our systematic review can provide some useful information for designing future trials. First, since evidence of the benefits of short term treatment with MMF is needed, studies comparing effects of long with short term treatment might be preferred in future. Second, trials with longer follow-up periods are required for the further study of the long term effects of MMF. Third, therapeutic effects comparing MMF with other immunosuppressive agents were reported in only a few studies and most of the patients were Asians. Therefore, large, multicenter clinical trials comparing the efficacy and safety of MMF and other immunosuppressive agents, particularly studies encompassing multiple ethnic groups, will be very valuable.

## Conclusion

Our subgroup analysis suggested that a relatively short course of MMF therapy may have enhanced potential therapeutic effects such as reducing proteinuria and lowering or stabilizing Scr levels in IgA nephropathy patients. However, this conclusion may only be tentatively drawn owing to the small sample size of the available published RCTs. No statistically significant difference has yet been found between MMF treatment and controls with regard to the need for renal-replacement treatment or 50% increase in Scr levels. High-quality RCTs with large sample sizes are needed to further define the efficacy and safety of MMF therapy in IgA nephropathy.

## Electronic supplementary material

Additional file 1: **Search strategy.** (DOC 22 KB)

Additional file 2: **PRISMA checklist.** (DOC 66 KB)

Additional file 3: **Risk of bias of the included studies judged by the review authors.** (DOC 39 KB)

Additional file 4: **Funnel plot.** (PDF 13 KB)

## Abbreviations

IgA: Immunoglobulin A
MMF: Mycophenolate mofetil
RCT: Randomized controlled trials
ESRD: End-stage renal disease
BP: Blood pressure
ACEI: Angiotensin converting enzyme inhibitor
ARB: Angiotensin receptor blocker
CBM: Chinese Biomedical Literature Database
CNKI: China National Knowledge Infrastructure
Scr: Serum creatinine
RR: Risk ratios
CI: Confidence intervals
MD: Mean difference
SMD: Standardized mean difference
CTX: Cyclophosphamide
LEF: leflunomide
CCB: Calcium channel blocker
eGFR: Estimated glomerular filtration rate.
