# Supplementary material for: Efficacy and safety of mycophenolate mofetil treatment in IgA nephropathy: a systematic review
Source: BMC Nephrol. 2014 Dec 5;15:193. doi: 10.1186/1471-2369-15-193 (PMC4267433; doi:10.1186/1471-2369-15-193)
Supplement: Supplementary file 1 — Additional file 1: Search strategy. (DOC 22 KB) [file 12882_2014_879_MOESM1_ESM.doc]

1. randomized controlled trial.pt
2. (randomized or randomised).ab,ti.
3. placebo.ab,ti
4. dt.fs.
5. randomly.ab,ti
6. trial.ab,ti
7. groups.ab,ti
8. or/1-7
9. exp animals/
10. exp humans/
11. 9 not(9 and 10)
12. 8 not 11
13. Exp IgA nephropathy/
14. IgA＄.tw.
15. Exp Berger’s disease/
16. Or/13-15
17. Exp mycophenolate mofetil/
18. Exp mycophenolate mofetil therapy/
19. Exp mycophenolic acid/
20. Exp MMF/
21. Exp MMF therapy/
22. Exp CellCep/
23. Exp cellcep therapy/
24. (mycophen＄ or cellcep).tw.
25. Or/17-24
26. 16 and 25
27. 12 and 26
