# Supplementary material for: Efficacy and safety of mycophenolate mofetil treatment in IgA nephropathy: a systematic review
Source: BMC Nephrol. 2014 Dec 5;15:193. doi: 10.1186/1471-2369-15-193 (PMC4267433; doi:10.1186/1471-2369-15-193)
Supplement: Supplementary file 2 — Additional file 2: PRISMA checklist. (DOC 66 KB) [file 12882_2014_879_MOESM2_ESM.doc]

| **Section/topic** | **#** | **Checklist item** | **Reported on page #** |
| --- | --- | --- | --- |
| **TITLE** | | |  |
| Title | 1 | Efficacy and safety of Mycophenolate Mofetil treatment on IgA nephropathy: a systematic review | 1 |
| **ABSTRACT** | | |  |
| Structured summary | 2 | Background,Method,Result,Conclusion | 2-3 |
| **INTRODUCTION** | | |  |
| Rationale | 3 | Introduction | 3-4 |
| Objectives | 4 | RCTs about MMF therapy in IgA nephropathy. | 4 |
| **METHODS** | | |  |
| Protocol and registration | 5 | protocol | *Additional file5* |
| Eligibility criteria | 6 | Study Selection criteria | 4-5 |
| Information sources | 7 | MEDLINE, EMBASE, Cochrane Library, CBM, and CNKI up to August 2014 | 4 |
| Search | 8 | relevant text words and Medical Subject Headings covering IgA nephropathy, IgA, GN, IgAGN, Berger’s disease, mycophenolate mofetil, mycophenolic acid, MMF, CellCept, controlled clinical trial, RCT, randomized controlled trial and drug therapy. | 4& *Additional file1* |
| Study selection | 9 | No language restriction, The search was limited to RCTs that assessed the effects of MMF therapy in IgA nephropathy and had a follow-up period of at least 6 months, | 4 |
| Data collection process | 10 | Data Extraction and Quality Assessment | 4-5 |
| Data items | 11 | - | - |
| Risk of bias in individual studies | 12 | Data Extraction and Quality Assessment | 4-5&*Additional file3* |
| Summary measures | 13 | Outcome measures | 5-6 |
| Synthesis of results | 14 | Statistical analysis | 6 |

Page 1 of 2

| **Section/topic** | **#** | **Checklist item** | **Reported on page #** |
| --- | --- | --- | --- |
| Risk of bias across studies | 15 | Data Extraction and Quality Assessment | 4-5& *Additional file3* |
| Additional analyses | 16 | Subgroup analysis | 5-6 |
| **RESULTS** | | |  |
| Study selection | 17 | Search results | 6-7 |
| Study characteristics | 18 | Characteristics of the included studies | 7&table1 |
| Risk of bias within studies | 19 | Study quality | 7-8 |
| Results of individual studies | 20 | Outcome | 8-10 |
| Synthesis of results | 21 | Outcome | 8-10 |
| Risk of bias across studies | 22 | Heterogeneity among studies | 7 |
| Additional analysis | 23 | subgroup analyses | 8-10 |
| **DISCUSSION** | | |  |
| Summary of evidence | 24 | Line201-215 | 10-11 |
| Limitations | 25 | Line259-263 | 13 |
| Conclusions | 26 | Conclusion | 14 |
| **FUNDING** | | |  |
| Funding | 27 | No | - |
