# Supplementary material for: Efficacy and safety of mycophenolate mofetil treatment in IgA nephropathy: a systematic review
Source: BMC Nephrol. 2014 Dec 5;15:193. doi: 10.1186/1471-2369-15-193 (PMC4267433; doi:10.1186/1471-2369-15-193)
Supplement: Supplementary file 3 — Additional file 3: Risk of bias of the included studies judged by the review authors. (DOC 39 KB) [file 12882_2014_879_MOESM3_ESM.doc]

**Additional file3**. Risk of bias of the included studies judged by the review authors

| Study | Randomisation method | Allocation concealment | Blinding: Participants | Blinding: Investigators | Blinding: Outcome assessors | Blinding: Data assessors | %Follow-up |
| --- | --- | --- | --- | --- | --- | --- | --- |
| Meas et al.2004 ^21^ | Unclear | Unclear | High | Unclear | NS | Unclear | 88 |
| Frisch et al.2005 ^22^ | Low | Low | Low | Low | Unclear | Unclear | 100 |
| Tang et al.2005 ^23^ | Unclear | Unclear | Unclear | Unclear | Unclear | Unclear | 100 |
| Chen et al.2002 ^20^ | Unclear | Unclear | Unclear | Unclear | Unclear | Unclear | 63 |
| Bao et al.2007 ^25^ | Unclear | Unclear | Unclear | Unclear | Unclear | Unclear | 81 |
| Zhao et al.2005 ^24^ | Unclear | Unclear | Unclear | High | Unclear | High | 100 |
| Liu et al. 2014^27^ | Low | Unclear | Unclear | Unclear | Unclear | Unclear | 100 |
| Liu et al.2010 ^26^ | Unclear | Unclear | Unclear | Unclear | Unclear | Unclear | 100 |

Low: low risk of bias; high: high risk of bias; Unclear: Unclear risk of bia
