# Supplementary figures and images for: Efficacy and safety of mycophenolate mofetil treatment in IgA nephropathy: a systematic review
Source: BMC Nephrol. 2014 Dec 5;15:193. doi: 10.1186/1471-2369-15-193 (PMC4267433; doi:10.1186/1471-2369-15-193)

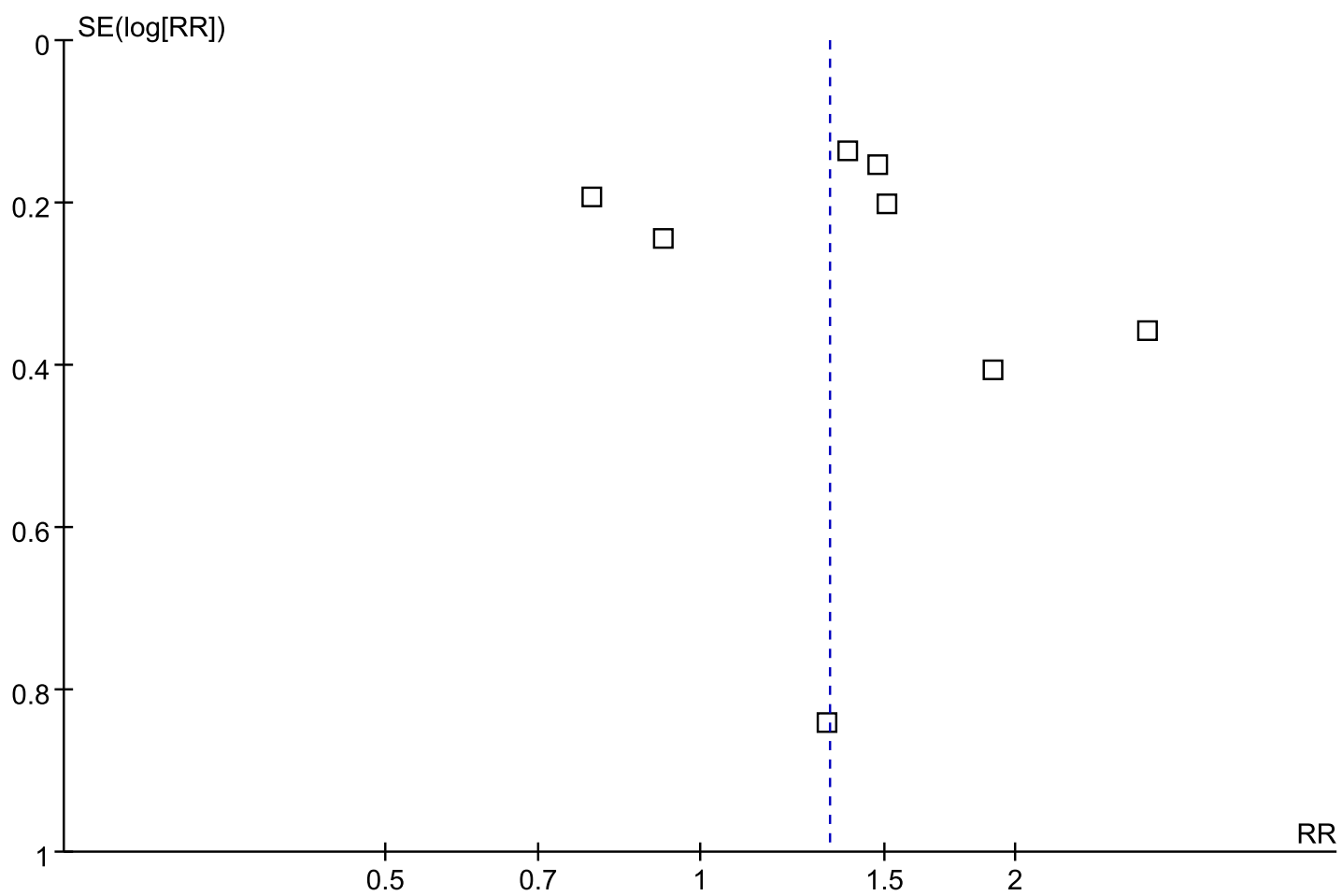

Supplement: Supplementary file 4 — Additional file 4: Funnel plot. (PDF 13 KB) [file 12882_2014_879_MOESM4_ESM.pdf]
